# Noise Suppression and Edge Preservation for Low-Dose COVID-19 CT Images Using NLM and Method Noise Thresholding in Shearlet Domain

**DOI:** 10.3390/diagnostics12112766

**Published:** 2022-11-12

**Authors:** Manoj Diwakar, Prabhishek Singh, Chetan Swarup, Eshan Bajal, Muskan Jindal, Vinayakumar Ravi, Kamred Udham Singh, Teekam Singh

**Affiliations:** 1 Computer Science and Engineering Department, Graphic Era Deemed to be University, Dehradun 248007, India; 2 School of Computer Science Engineering and Technology, Bennett University, Greater Noida 201310, India; 3 Department of Basic Science, College of Science and Theoretical Studies, Saudi Electronic University, Riyadh-Male Campus, Riyadh 13316, Saudi Arabia; 4 Department of Computer Science and Engineering, Amity School of Engineering and Technology, Amity University, Noida 201303, India; 5 Center for Artificial Intelligence, Prince Mohammad Bin Fahd University, Khobar 34754, Saudi Arabia; 6 Department of Computer Science and Information Engineering, National Cheng Kung University, Tainan 701, Taiwan; 7 School of Computer Science, University of Petroleum and Energy Studies, Dehradun 248007, India

**Keywords:** COVID, CT image denoising, thresholding, World Health Organization

## Abstract

In the COVID-19 era, it may be possible to detect COVID-19 by detecting lesions in scans, i.e., ground-glass opacity, consolidation, nodules, reticulation, or thickened interlobular septa, and lesion distribution, but it becomes difficult at the early stages due to embryonic lesion growth and the restricted use of high dose X-ray detection. Therefore, it may be possible for a patient who may or may not be infected with coronavirus to consider using high-dose X-rays, but it may cause more risks. Conclusively, using low-dose X-rays to produce CT scans and then adding a rigorous denoising algorithm to the scans is the best way to protect patients from side effects or a high dose X-ray when diagnosing coronavirus involvement early. Hence, this paper proposed a denoising scheme using an NLM filter and method noise thresholding concept in the shearlet domain for noisy COVID CT images. Low-dose COVID CT images can be further utilized. The results and comparative analysis showed that, in most cases, the proposed method gives better outcomes than existing ones.

## 1. Introduction

The novel coronavirus disease 2019 (COVID-19) emerged in early December 2019 in the Hubei province of the People’s Republic of China, and it was caused by the virus Severe Acute Respiratory Syndrome Coronavirus 2 (SARS-CoV-2). It rapidly spread to various countries and developed into a global outbreak. The World Health Organization (WHO) declared the outbreak as a global pandemic on 11 March 2020 [1,2]. This also led to a massive strain on healthcare systems worldwide. 

While CT scan imagery might be effective in coronavirus detection, it has certain limitations when it comes to continuous use and its subsequent hazardous side effects. The detection of COVID-19 can be performed by identifying lesions in scans, namely, ground-glass opacity, consolidation, nodules, reticulation, or thickened interlobular septa, and lesion distribution [3,4], but in the early stages, due to the nascent development of lesions and limited use of high dose X-rays, identification is difficult. These symptoms, when not detected early, multiply the spread not only for the victim, but also for others around the victim. The dilemma here is that, for early diagnoses of infection, doctors require either the continuous use of X-rays or the use of high-dose X-rays, both of which lead to hazardous side effects such as vomiting, bleeding [5,6,7,8,9], fainting, hair loss, and the loss of skin and hair and cancerous cells [9,10,11,12,13,14,15]. This leaves us to use low-dose X-rays for CT scans, which give us scans corroded by noise and disturbance. Thus, deliberating the above, the use of high-dose X-rays may create further complications for a patient who may or may not be infected with coronavirus [16,17,18]. Conclusively, the only way to prevent patients from side effects consistent with high-dose X-rays while diagnosing the presence of coronavirus early is using low-dose X-rays to generate CT scans, then implementing a robust denoising algorithm to the scans [19,20,21,22,23]. As explained above, the importance and need for denoising have developed more than ever. Moreover, image segmentation for the classification and identification of the type of lesion is also a substantial task in the diagnosis procedure. There are different methods for it, and various researchers have contributed to the cause [24,25,26,27,28,29].

Medical image denoising [30,31,32,33,34,35,36] is the process of removing corrosive and corrupting elements from the image in order to obtain an image of superior clarity and quality. While there are multiple techniques used to denoise images, they are graded into two major purviews, namely spatial techniques such as stationary multiplicative speckle model filters (SMSM), non-stationary multiplicative speckle model filters (NSMSM), among others, and transform techniques such as threshold-based techniques, Bayesian techniques, directional transform techniques, non-local means (NLM), block-matching 3D (BM3D), and others, each having variegated techniques with respective advantages and disadvantages [37]. Another robust technique in the field of image processing for handling tasks such as image denoising and image segmentation in shearlet transform consists of a multi-scale framework for the multi-dimensional representation of images. Recently, shearlet transform is being implemented in variegated domains; for example, the study [38] used optimized threshold shearlet transform for denoising microscopic data by eliminating random noise. Thresholding is performed for shearlet coefficients by shrinking the same the microscopic data random noise that is distributed unevenly. The novelty of the method lies in the use of adaptive thresholding coefficients, which are obtained by the use of the adjustment factor for each fundamental sub-band individually [39].

Another state-of-the-art denoising technique implements a shearlet transform that amalgamates the same, with an oriented second-order partial equation, to remove multiplicative speckle noise in electronic speckle pattern interferometry (ESPI) fringe patterns. The novel proposition of this study lies in the use of shearlet transform in ESPI fringe patterns with the aim to denoise. It leverages the concept that the spatial-domain filtering method SOOPDE and shearlet transform are symbiotic to each other on the grounds of mutual benefit [40]. This study leveraged the unique ability of shearlet transform to handle multi-modal data at a multi-scale level such as a wide range of orientations, complex geometric figures, etc. It aimed to gauge the performance of shearlet transform for denoising and segmentation on the grounds of space-time complexity for a set of GPUs by implementing 2D and 3D on standard GPU and proposed accelerated GPU. Preserving edges and the texture of images when denoising is a challenging task. Non-subsampled shearlet transform (NSST) can perform a tenable job, but amalgamating with a twin support vector machine can provide better results, as conducted in the presented study [41]. Primarily, it divides the noisy image into multiple sub-bands oriented to NSST, later using a twin support vector for feature detection and denoising [42].

For a similar study that implemented NSST for image denoising, its novelty lied in amalgamating sparse representation classification with NSST. This study followed a multi-step procedure. NSST coefficients are primarily classified into noise or edge coefficients using spars representation via variable splitting and augmented Lagrangian (SUnSAL) classifiers, posting which noise coefficients are denoised by the shrink method with adaptive Bayesian threshold. Finally, inverse NSST reverses the coefficients to obtain the denoised image [43,44,45,46,47,48,49,50]. A similar technique that implements shearlet transform via signal detecting operation by using a state-of-the-art proposed strategy that uses the correlation with energy distribution of tri-component microseismic signals shows substantial performance both on stimulated and real noisy data sets [51,52]. Optimizing shearlet transformation with fellow wavelet techniques is common, but it can be performed by manipulating the coefficients shown by the presented work [53]. This study shows a groundbreaking increase in the performance of shearlet transform by manipulating its constraints of residual coefficients.

This study proposes a novel low-dose CT image denoising technique by amalgamating two state-of-the-art methodologies, the NLM filter and Bivariant thresholding, that provides superior results to the classic NLM filter, which suppresses sharp noise and thresholding, which works on edge preservation. The presented research framework evaluated and analyzed the results obtained in imageries via qualitative and quantitative analysis and graphical and statistical means. The rest of the paper is organized as follows: A brief overview of methods such as NLM and shearlet transform are given in Section 2. In Section 3, the proposed methodology is described. In Section 4, results are analyzed and discussed. Finally, the conclusion is drawn in Section 5.

## 2. Preliminaries

In this section, some major concepts are discussed that were utilized in the proposed methodology.

### 2.1. NSST or Non-Subsampled Shearlet Transform

Through the use of decomposition and the preservation of translation invariance, NSST is able to accomplish multi-scale and multi-directional transformations while avoiding the Gibbs phenomenon. The pseudo-grid is processed using a two-dimensional Fourier transform before being passed through a 1D sub-band filter. As a result, essential sampling can be eliminated. Due to its ability to adapt to the diverse geometrical properties of multi-dimensional and multi-scale data, NSST has an edge over other transform methods.

(1) $$NSLP_{j+1}=A_{j}f=\left(Ah_{j}^{1}\prod_{k=1}^{j-1}Ah_{k}^{0}\right)f$$

Primarily, the low-pass image and the high-pass image are obtained by decomposing the original image. The coefficient for the pseudo grid is obtained by calculation, which is then used to obtain frequency-domain coefficients by bandpass filtering. Finally, non-subsampled shearlet transform coefficients are obtained by implementing an inverse transformation of the fast Fourier transform (FFT) on the pseudo grid.

For p > 0, q ∈ R, t ∈ R^2^, the shearlets can be expressed as:

(2) $$\Psi_{p.q.f}=|defF_{p.q}{|}^{\frac{1}{\Delta }}\Psi \left(F_{pq}^{-1}\left(x-f\right)\right)$$

where $\;F_{p,q}=B_{q}A_{p}=(\begin{array}{cc} p & \surd pq\\ 0 & \surd p \end{array})$, $A=\left(\begin{array}{cc} p & 0\\ 0 & \sqrt{p} \end{array}\right)$, $B_{q}=\left(\begin{array}{cc} 1 & q\\ 0 & 1 \end{array}\right)$.

Only the shear matrix controls the shearlet’s direction. As a result, the shearlet transform depends on the scale (p), the orientation (q), and the position. Each *f* ∈ L^2^ (R^2^) can be recovered by:

(3) $$f=\int_{R^{2}}^{}\int_{-\infty }^{+\infty }\int_{0}^{+\infty }<f,\;\Psi_{p.q.f}>\Psi_{p.q.f}\frac{dp}{p^{3}}dqdt$$

Because of its mathematical basis, the discrete shearlet transform can be effectively represented in a variety of contexts. The discrete implementation of the shearlets transform offers the ability to take into account functions with multiple dimensions. With this consideration, p = 2 – 2J, q = −L with J, t = K ∈ Z2, and L ∈ Z. The discrete shearlet transform is expressed as:

(4) $$\Psi_{p.q.f}=|\det\;A_{0}{|}^{\frac{J}{2}}\Psi \left(B_{0}^{L}A_{0}^{L}x-K\right)$$

where $A_{0}=\left(\begin{array}{cc} 4 & 0\\ 0 & 2 \end{array}\right)$, and $B_{0}=\left(\begin{array}{cc} 1 & 1\\ 0 & 1 \end{array}\right)$.

For each $\in L^{2}\;\left(R^{2}\right)$, the formula can be reproduced with the characteristic Ψ, as shown below:

(5) $$f=\sum_{J,L\;\in \;Z,K\;\in \;Z2\;}^{}\langle f,\;\psi_{J\cdot L.K}\rangle \psi_{J\cdot L.K}$$

### 2.2. Non-Local Mean (NLM)

NLM effectively preserves the variegated regions of multiple types of textures while cleaning the input image. While other filters deliberate the mean of neighboring pixels of the target image, i.e., pixels surround target pixels, NLM deliberates the mean of all pixels in the target image. However, the weights depend upon the similarity of pixels, or simply put, the higher the similarities higher the weights are.

(6) $$NL\left[v\right]\left(i\right)=\sum_{j\in I}w\left(i,j\right)v\left(j\right)$$

This approach results in a lesser degree of distortion of the denoised pixel from the original when compared to other methods. The mean of all pixels across is taken as a whole using a Gaussian window, and the value of a particular pixel is evaluated based on its position in the image, up to a range of values determined by the mean pixel value of said image.

## 3. Proposed Methodology

**Figure 1:**
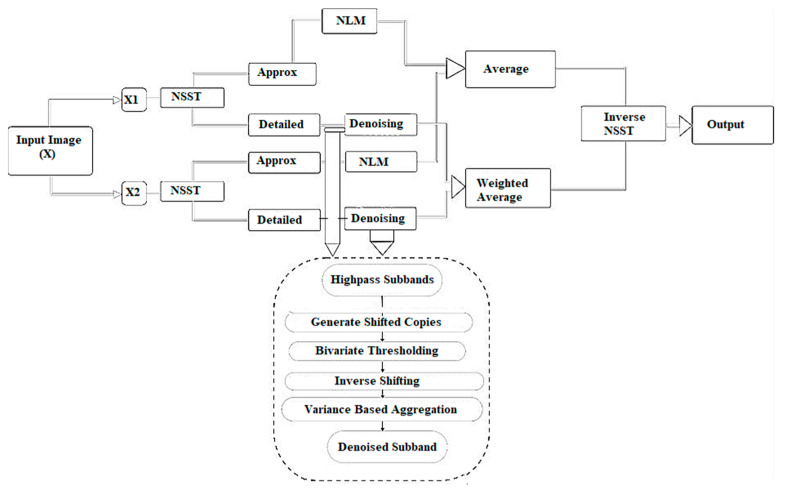
Proposed algorithm.

With the assumption that CT images are noisy due to additive white Gaussian noise, a new method was proposed to remove the noise from CT images. Figure 1 shows the complete overview of the proposed framework. This noise predominantly attacks mainly on high frequencies, although its effect on low frequencies is non-trivial. In the proposed method, the noise removal process is included so that noise is suppressed effectively and the edges are preserved at the same time. In the proposed methodology, the characteristics of the NLM filter are utilized, and a novel thresholding function was proposed for noise suppression and edge preservation.

### Proposed Thresholding Function

Let the CT image be noisy, which can be denoted as:

*X*(*p*,*q*) = *Y*(*p*,*q*) + *n*(*p*,*q*) (7)

where *X(p,q)* is a noisy CT image, *Y* is a clean CT image, and n is an additive Gaussian noise.

This is used in the filtration of high-frequency bands because it is a derivative of bilateral thresholding. Taking the position of a coefficient of a wavelet function and substituting it with its parent of a higher scale forms the background of this function. Assuming that *w*_1*k*_ is the child of *w*_2*k*_, where *w*_1*k*_ is the kth complex wavelet coefficient at the same position as that of *w*_1*k*_, then we can express them as:

(8) $$\begin{array}{c} y_{1k}=w_{1k}+\eta_{1k}\\ y_{2k}=w_{2k}+\eta_{2k} \end{array}$$

where *y*_1*k*_ and *y*_2*k*_ are noisy complex wavelet coefficients, η_1*k*_ and η_2*k*_ are additive noise coefficients, and *w_k_* = (*w*_1*k*_, *w*_2*k*_), *y_k_* = (*y*_1*k*_, *y*_2*k*_), and η*_k_* = (η_1*k*_, η_2*k*_). From this equation, after a bit of mathematical manipulation, the bivariate shrinkage function or thresholding can be expressed as:

(9) $$w{ˆ}_{1k}=\frac{\;\sqrt{y_{1k}^{2}+{\;y}_{2k}^{2}\;}–{\;\lambda }_{k}}{\sqrt{y_{1k}^{2}+{\;y}_{2k}^{2}\;}}*y_{1k}$$

The function is defined as:

(10) $$g{\left(a\right)}_{*}=\left\{\begin{array}{c} a\;\;\;\;\;\;\;\;\;\;\;\;\;\;\;\;\;\;if\;a>0\\ 0\;\;\;\;\;\;\;\;\;\;\;\;\;\;\;otherwise \end{array}\right.$$

In the suggested thresholding equation, the multi-variate function is modified by the definition of process noise with the SURE-LET technique. Therefore, it can be expressed as:

(11) $$z_{c}=y_{c}-{\hat{w}}_{c}$$

(12) $$\theta \left(y_{c}\right)=d_{1}{\hat{w}}_{c}+d_{2}z_{c}$$

where the weight values that should be optimized to achieve the best outcomes are *d*_1_ and *d*_2_.

It can be expressed as:

(13) $$\theta \left(y_{c}\right)=\left(d_{1}-d_{2}\right){\hat{w}}_{c}+d_{2}y_{c}$$

Let $a_{1}=d_{2},\;a_{2}=d_{1}-d_{2},\;W_{c}^{1}={\hat{w}}_{c}$, and $W_{c}^{2}=y_{c}$, and then the above equation can be expressed as:

(14) $$\theta \left(y_{c}\right)=a_{1^{W^{\wedge }k1}}+a_{2^{W^{\wedge }k2}}$$

where the parameter $a_{c}\;\left(1\leq c\leq 2\right)$ is linear.

It is possible to re-design the equation above as:

(15) $$\theta \left(y_{c}\right)=\sum_{s=1}^{2}a_{s}\varphi_{s}\left(y_{c}\right)$$

A thresholding function is proposed for the above linear function, as follows:

(16) $$\theta \left(y_{\mathrm{new}\_c}\right)=\left\{\begin{array}{ll} 0, & \mathrm{if}\;\theta \left(y_{c}\right)<0\\ \theta \left(y_{c}\right)\;, & \mathrm{Otherwise} \end{array}\right.$$

A composition of different estimates of noise-unfastened coefficients is the suggested thresholding function. For the best denoising outcomes, the best *a_k_* weight values must be optimized. An unbiased mean-square-error (MSE) estimator is known to optimize weight values, followed by the SURE-LET method. It can be conveyed as:

(17) $$\varepsilon =\frac{1}{MN}\sum_{c=1}^{MN}\left(\theta^{2}\left(y_{c}\right)-2y_{c}\theta \left(y_{c}\right)+2\sigma^{2}\theta \left(y_{c}\right)\right)+\sum_{s=1}^{MN}w_{c}^{2}$$

To estimate the parameter $a_{\langle }$, only $\varepsilon =\frac{1}{MN}\sum_{}^{}(\theta^{2}\left(y_{\langle }\right)-2y_{\langle }\theta \left(y_{\langle }\right))$ MSE for $a_{s}$ can be expressed as:

(18) $$\sum_{f=1}^{4}P_{s.f}a_{\zeta }-Q_{s}=0$$

where $P_{s.f}=\left[\varphi_{s}\left(y\right)\varphi_{\zeta }\left(y\right)\right]$ and $Q_{s}=\left[y\varphi_{s}\left(y\right)-\sigma^{2}\varphi_{s}\left(y\right)\right]$. The above equation can be solved by the linear method: $a_{\zeta }=P_{s.f}^{-1}Q_{s}$

Figure 1 successfully illustrates the denoising scheme followed in the presented framework. It is evident that low-dose CT images have different levels of accuracy due to noise intensity and pattern that vary considerably; thus, two images were taken to generate a more accurate result. It must be noted that, although two images may require two CT scans, the total radiation of the two combined would still be considerably less than the radiation exposure from a normal CT scan.

The summary of the proposed work is shown in Algorithm 1:
**Algorithm 1:** CT image denoising.Step 1: Firstly, read input noisy CT image. Step 2: Non-subsampled shearlet transform is applied to both noisy images, which divides the image into two parts:
 a. Approximation part (H)
 b. Detailed part (D)
Step 3: Apply k and l directional circular shift to obtain n high-frequency sub-bands of both input images.     $\;H\prime {\left(k,l\right)}_{s^{y}}=\mathrm{circular}\_\mathrm{shift}\;\left\{H{\left(k,l\right)}_{s},\left[k_{\mathrm{shift}},l_{\mathrm{shift}}\right]\right\}$ Step 4: Perform NLM filter on both approximation part. Step 5: Perform average operation on the outcomes of Step 4. Step 6: For all levels in high-frequency sub-bands of both input images: (a) Calculate the threshold value (b) Apply shrinkage rule using Equation (16) Step 7: To obtain an enhanced high-frequency sub-band, calculate the weighted average based on patch variance on the outcome of Step 6:         $\hat{H}{\left(k,l\right)}_{s}=\sum_{y=1}^{n}\alpha_{s^{y}}.H\prime \prime {\left(k,l\right)}_{s^{y}}$
where $\alpha_{s^{y}}$ = $\frac{{\mathrm{var}}^{-1}\left(X_{w,s^{y}}^{n}\right)}{\sum_{y=1}^{n}{\mathrm{var}}^{-1}\left(H_{w,s^{y}}^{n}\right)}$, var^−1^ (.) represents the inverse of threshold, and H(k,l)s is the final threshold value. Step 8: To obtain the final output image, perform the inverse of the circular shift using the outcome of Step 5 and Step 7:            $H\prime \prime \left(k,l\right)\mathrm{sy}=\mathrm{circular}\_\mathrm{shift}\left\{\hat{H}\prime {\left(k,l\right)}_{s^{y}},\left[-k_{\mathrm{shift}},-l_{\mathrm{shift}}\right]\right\}$

## 4. Results and Discussion

**Figure 2:**
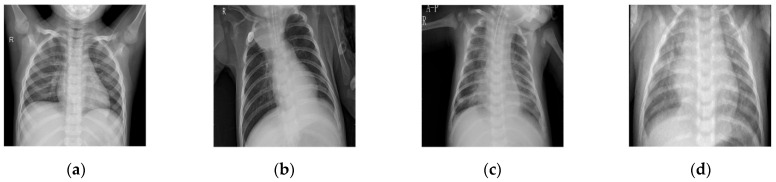
COVID-19 reference CT image dataset; (**a**) CT1 image; (**b**) CT2 image; (**c**) CT3 image; (**d**) CT4 image.

The experiments were carried out in Matlab 2018 running on a system comprising an i5 8250 with 8 GB of RAM running 64-bit Windows 10 as the operating system. All images used were of dimensions 512 × 512 pixels, as illustrated in Figure 2, which were taken from the Cornell open-access library dataset.

In the proposed method, a thresholding function was performed in detail parts of NSST domain. In the approximation part of NSST, the NLM filter was performed. The denoising process was performed using patch-wise circular shifting. To show the significance of the proposed method, performance metrics (PSNR, SSIM, DIV, and ED) were estimated without circular shifting and with circular shifting using different patches. The average values (87 images) of the performance metrics are shown in Table 1. Here, it can be clearly analyzed that the best values were given by the proposed method, with circular shifting using 5 × 5 patches. Hence, in our proposed method, 5 × 5 patches were used to evaluate the result analysis.

### 4.1. Comparative Analysis

**Figure 3:**
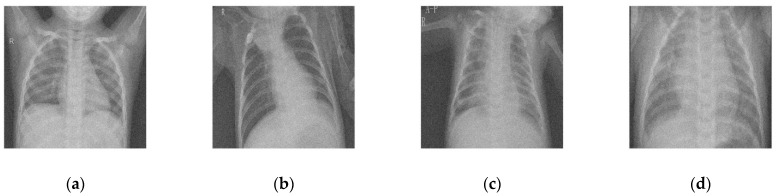
COVID-19 noisy CT image dataset corresponding to Figure 2; (**a**) Noisy CT1 image; (**b**) Noisy CT2 image; (**c**) Noisy CT3 image; (**d**) Noisy CT4 image.

**Figure 4:**
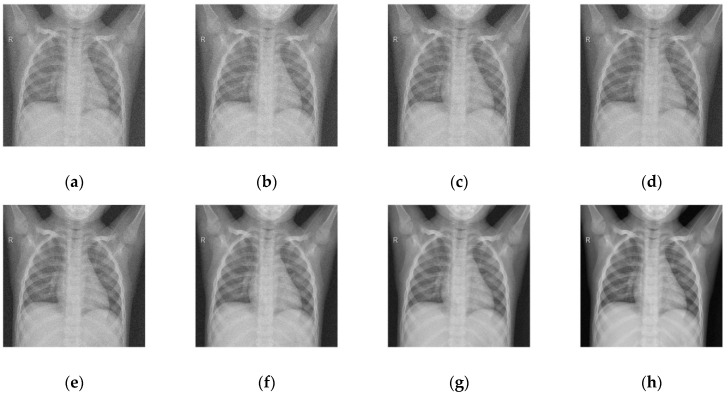
Denoised COVID-19 CT image 1 from the (**a**) [10], (**b**) [11], (**c**) [12], (**d**) [13], (**e**) [14], (**f**) [11], (**g**) [16], and (**h**) proposed frameworks, respectively.

**Figure 5:**
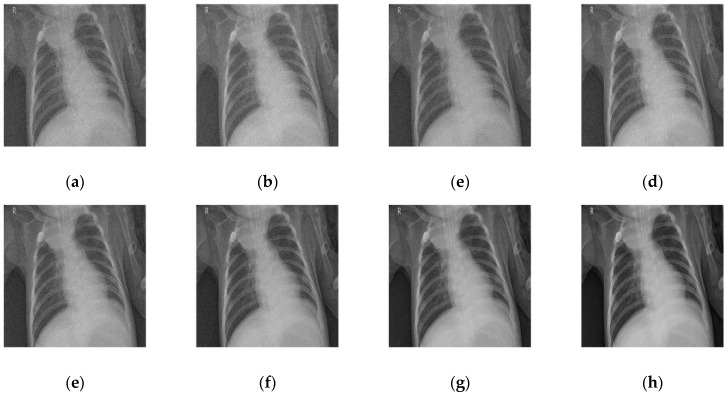
Denoised COVID-19 CT image 2 for the (**a**) [10], (**b**) [11], (**c**) [12], (**d**) [13], (**e**) [14], (**f**) [11], (**g**) [16], and (**h**) proposed frameworks, respectively.

**Figure 6:**
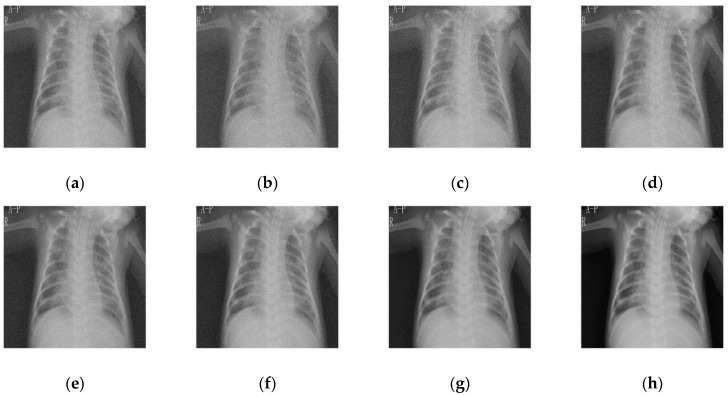
Denoised COVID-19 CT image 3 from the (**a**) [10], (**b**) [11], (**c**) [12], (**d**) [13], (**e**) [14], (**f**) [11], (**g**) [16], and (**h**) proposed frameworks, respectively.

**Figure 7:**
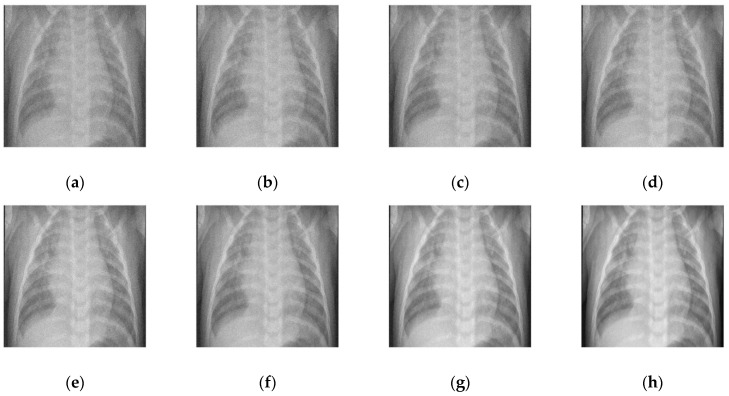
Denoised COVID-19 CT image 4 for the (**a**) [10], (**b**) [11], (**c**) [12], (**d**) [13], (**e**) [14], (**f**) [11], (**g**) [16], and (**h**) proposed frameworks, respectively.

**Figure 8:**
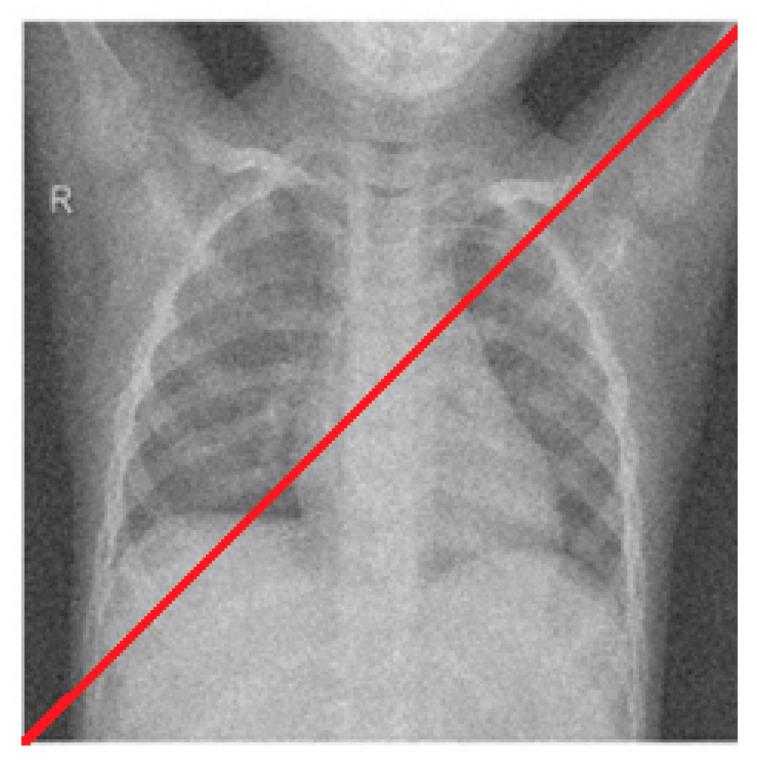
The line used for intensity profiling in Image 1 for all the frameworks.

To maintain consistency, all the Gaussian noise present in the images was artificially applied. Gaussian noise was added to all the images at different intensities, with sigma values between 30 and 5. The noisy images are shown in Figure 3. Figure 4, Figure 5, Figure 6 and Figure 7 show the results of the denoising process, as performed by the different competing algorithms that were compared. Figure 5 shows the denoised output from all eight different frameworks when applied on the first image from Figure 1, respectively. Similarly, Figure 6, Figure 7 and Figure 8 show the results of the denoising process for the case of images 2, 3, and 4 from Figure 2 as outputted by the frameworks [10,11,12,13,14,15,16], respectively.

As shown in Figure 4, Figure 4a, corresponding to [10], shows the least feasible result of the group. The edges were not preserved, the contrast was altered, and rogue pixels were introduced, creating noise. Although the result was better than the noisy image, the loss of detail and the excessive fuzziness take away from its ability to be useful in real-world applications. Figure 4b, corresponding to [11], shows slightly better results, although the overall quality was still much lower than the quality of the other techniques. This framework introduces less noise but even more blur than [10]. The details of [12] and [13] are very incorrigible, with visually noticeable noise generated from the region with intermediate grey values.

In Figure 5, the frameworks deal with images that need more contrast preservation and heterogeneous integrity. Figure 5a, corresponding to [10], gave the worst output, with no noticeable improvement from the noisy image. Figure 4b,c shows the results of [12] and [13]. The best results demonstrated by Figure 5g,h correspond to [16] and the proposed method. No doubt, the proposed system came out on top; however, [16] came pretty close in terms of contrast preservation. Nothing special or noteworthy was illustrated, showing a generally average performance with a slight loss in the intensity of black pixels in all of [11,14,15].

While all the frameworks performed very close to each other for this image, [10] showed a high level of fuzziness or blur introduced as denoising artifacts by the overcorrection of the image. The [11] framework showed good edge preservation, with the contrasts almost accurate to the original, but the presence of increased noise in the background degraded the overall quality of the image. Similarly, [12] sufficiently removed noise with proper clarity and no added noise; the detail preservation was actually inferior to [11], especially in heterogeneous noisy areas. The [13] framework changed the background and increased the overall pixel value of the image by giving pixels that were black a lower color value. Amongst all the methods tested, [14] had the most inferior edge preservation results, introducing noise at the edges of the images, particularly when there was a high contrast between the subject and the background, which further corroded the image quality. Both [15,16] performed very well, with almost negligible performance between each other and the proposed methodology.

The outcome comparison of the fourth and final image is shown in Figure 7. The quantity of blur pertaining to the white noise introduced in [10] made the image as bad as the noisy image. Almost no improvement is seen, which implies that scans with regions dense in muscle will absolutely not work with [10]. Ref. [11], shown in Figure 7b, had better contrast and slightly better edge detection, although it was still way off from the original or even its competing denoised counterparts. Figure 7c, corresponding to [13], performed a satisfactory job, but it can be observed that the contrast was reduced all throughout the image, with the bones getting darker and the background getting lighter. Figure 7e,f shows nothing noticeably special. Satisfactory edge preservation, contrast maintenance, and noise suppression were conducted as expected. Figure 7g, the outcome from [15], was deemed as the best among the older techniques, coming closest to the original after the proposed technique. The only difference between it and the proposed framework was the combination of increased sharpness and slightly better contrast that is clear from the pattern of the spine in the two images. Figure 7g is less sharp, and the individual vertebrae are harder to distinguish, while Figure 7h is both whiter and easier to discern.

**Figure 9:**
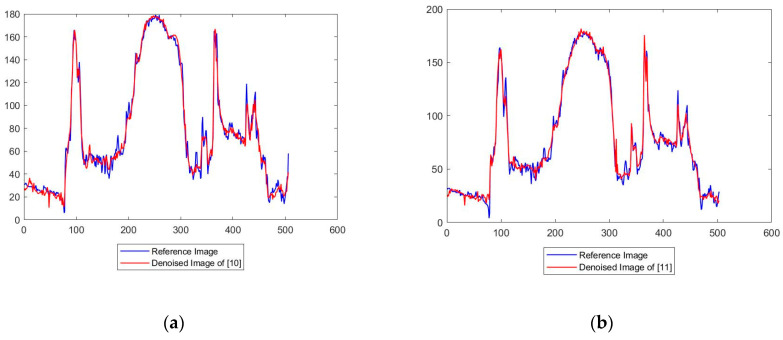

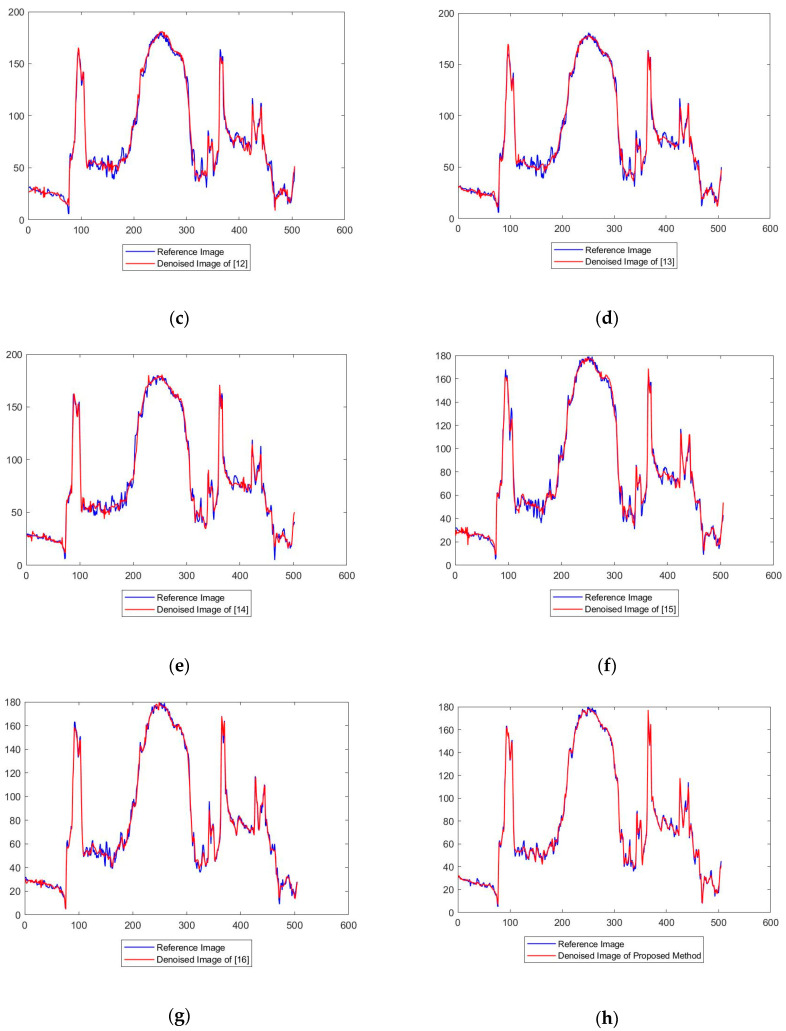
Intensity profiles of the original image against the [10,11,12,13,14,15,16] framework and the proposed framework, respectively; (**a**) Intensity profile between reference image and denoised image [10]; (**b**) Intensity profile between reference image and denoised image [11]; (**c**) Intensity profile between reference image and denoised image [12]; (**d**) Intensity profile between reference image and denoised image [13]; (**e**) Intensity profile between reference image and denoised image [14]; (**f**) Intensity profile between reference image and denoised image [15]; (**g**) Intensity profile between reference image and denoised image [16]; (**h**) Intensity profile between reference image and denoised image of proposed method.

Figure 8 shows a line used for intensity profiling in CT image for the analysis of all the frameworks. Figure 9 shows the intensity profiles of the original image against the [10,11,12,13,14,15,16] frameworks and the proposed framework, respectively. From Figure 8 and Figure 9, certain observations about the frameworks were made. While all the frameworks performed reasonably well, [10] showed a high level of fuzziness or blur introduced as denoising artifacts by the overcorrection of the image. The [11] framework showed good edge preservation, with the contrasts almost accurate to the original, but the presence of increased noise in the background degraded the overall quality of the image. Similarly, [12,13] performed sufficient removal of the noise with proper clarity and no added noise; the detail preservation was actually inferior to [11], especially in heterogeneous noisy areas. The [14] framework had the most inferior edge preservation results amongst all the methods tested, introducing noise at the edges of the images, particularly when there was a high contrast between the subject and the background, which further corroded the image quality. Both [15,16] performed very well, with almost negligible performance between each other and the proposed methodology.

The proposed framework was found to be supplanting all the other frameworks, scoring better in all aspects, including but not limited to edge preservation, noise suppression for both heterogeneous and contrasting areas of the input image, detail preservation, and overall similarity to the original image. The heterogeneous noise removal was good, with equally good contrast preservation to go with it. Thus, it can be confidently said that the proposed framework competed with and outperformed the other state-of-the-art frameworks in almost all aspects of speckle image denoising in medical CT scan images.

### 4.2. Performance Metrics

*SSIM*: The structural similarity index is a full reference metric used to quantify image degradation by comparing two windows, *X* and *Y,* to the original and processed image. This method works in a manner similar to humans and, hence, can be taken as a crude estimate close to what a human would grade the image. The original image is required for the use of this metric, the formula for which is given below:

(19) $$SSIM\left(x,y\right)=\frac{\left(2\mu_{x}\mu_{y}+C_{1}\right)\left(2\sigma_{xy}+C_{2}\right)}{\left(\mu_{x}^{2}+\mu_{y}^{2}+C_{1}\right)\left(\sigma_{x}^{2}+\sigma_{y}^{2}+C_{2}\right)}$$

where μ_x_, μ_y_ are the mean values of noisy and denoised images, respectively. *C*_1_ and *C*_2_ are the constant values; σ is the variance of the respective image.

*PSNR*: Peak signal-to-noise ratio, as is evident from the name, is the ratio between the total power, or original image in this case, to the power of the noise responsible for declining the image quality. It is also a full reference metric, and the formula is:

(20) $$PSNR=10\log_{10}\left(\frac{IMS}{MSE}\right)$$

where IMS is the image size in m*n pixels, and *MSE* is the mean square error calculated as: $MSE=\frac{1}{N}\sum_{j=0}^{N-1}{\left(X-Y\right)}^{2}.$

Entropy Difference (*ED*): Entropy is the randomness or chaos in a system. In the image, it is the chaos present, i.e., the difference from the original source. The entropy difference is the mean of the Sharon entropy (*SE*) between the clean and denoised image.

(21) $$ED=SE\left(X\right)-SE\left(Y\right)$$

Difference in Variance (*DIV*): Performance measure by variance (Var) used in statistics.

(22) $$DIV=1-\frac{VAR\left(X\right)}{VAR\left(Y\right)}$$

Table 2 shows the average of all the PSNR and SSIM values of all the images that were tested. The testing performed on a sample set of 90 images was enough to normalize and negate the impact of any bias that a technique might have for one particular image, whilst increasing the legitimacy of this technique. The *Σ* symbol corresponds to the value of sigma, which defines the amount of noise introduced in the images. The higher the value, the greater the noise introduced in the image and the lower the quality of the output image even after denoising. In this table, the first column shows the different frameworks, and the next four columns show the values of PSNR corresponding to the particular framework at a particular noise level. This is why the values kept decreasing as the value of *Σ* kept increasing. In almost all the cases, the values generated by the different frameworks were close to each other, with differences noticeable. Except for *Σ* = 30, the proposed framework was superior to all other frameworks by a noticeable margin of 0.4–0.9. This is greater than the average difference between results amongst the other frameworks, proving the efficacy of this technique. Overall, by comparing on the basis of SSIM and PSNR, it was observed that [15] was the best among the existing techniques, and although the proposed technique is better than [15], the difference was small. The [11] framework showed the most consistency, with a graceful decline in scores with the increase of noise levels. The other techniques, [10,12,13,14,16], performed reasonably without anything notable worth mentioning.

Similar to Table 2, Table 3 shows the ED and DIV values of the denoised images generated from the frameworks at different noise levels. Unlike the previous techniques, these values increased with the increase in noise and subsequently for the dissimilarity between images. In this case, [14] was the most volatile and shows the largest variation with a large range and huge jumps in scores with the increase in noise. The [16] framework scored relatively well, coming very close to the proposed framework at lower noises but rapidly lost its performance with an increase in noise. The [10,11] frameworks had the lowest scores overall, which is in support of the observations made in the qualitative analysis. The [13] framework had an average set of scores, neither too low to be comparable to the proposed framework nor too high to be the worst of the set. The [12] framework’s ED value came closest to the proposed framework at the lowest noise level but showed no significant score in the rows pertaining to the higher noise values. Overall, the proposed framework beat all the systems, similar to its superior performance seen in Table 1. The DIV score of 0.02 at *Σ =* 10 is an order of magnitude better than the other competitions.

A total of 112 experts pertaining to the domain of image processing were consulted for their opinion on the quality of the image and, therein, their judgment on the performance of the algorithms. Table 4 shows the average of the scores granted by the experts to the various techniques in various aspects or features used in tandem with image denoising quality measurement. The scores range from 1 to 3, with 3 denoting the best performance. As seen in the table, the proposed framework garnered high approval in most of the fields, according to the experts. This is a clear demonstration that adds weight to the validity of the framework.

## 5. Conclusions

This study presented a novel hybrid denoising framework for low-dose CT images that combined non-subsampled shearlet transform and bivariate thresholding to obtain pristine results that surpassed the state-of-the-art techniques on the grounds of edge preservation, feature and detail preservation, denoising contrast, and heterogeneous area of the image, and it prevented the creation of artifacts during the process of denoising. The results obtained were validated by comparing the proposed framework with the latest techniques in the domain via qualitative analysis and quantitative analysis. Reference image indexes, namely, PSNR, SSIM, entropy difference, and variance difference of the proposed and state-of-the-art-methods, were compared to support the quantitative analysis. Similarly, the qualitative analysis was supported by the comparison of image intensity profiles between the original and denoised images. The presented study addressed the dilemma of using high-dose X-rays that provide noise-free images but have multiple deleterious effects versus using low-dose CT images that are corrupted by noise but have comparatively very few or negligible side effects. The presented study provided noise-free CT images that aid COVID-19 diagnoses without creating any salutary hazards to the patient due to X-rays, thereby being applied widely in clinical medical image capturing, classification, and diagnosis of coronavirus.

## Figures and Tables

**Table 1:** Average results (87 images) of performance metrics (PSNR, SSIM, DIV, and ED).

	Proposed Method (without Circular Shifting)		Proposed Method (with Patch-Wise Circular Shifting)							
	PSNR	SSIM	PSNR				SSIM			
Noise Variance			3 × 3	5 × 5	7 × 7	9 × 9	3 × 3	5 × 5	7 × 7	9 × 9
10	28.01	0.7408	28.3	29.4	28.8	28.5	0.7633	0.7855	0.7534	0.7432
20	26.13	0.6763	26.5	27.4	27.6	26.2	0.6933	0.7011	0.6922	6823
30	24.21	0.6024	24.6	25.7	25.5	24.7	0.6124	0.6321	0.6211	0.6121
40	22.03	0.5732	22.3	23.8	23.9	22.2	0.5911	0.6062	0.5982	0.5827
	ED	DIV	ED				DIV			
10	0.8131	0.5741	0.4232	0.3231	0.3123	0.4341	0.4402	0.3132	0.4713	0.4928
20	1.9312	1.6742	1.7542	1.0341	1.1212	1.3312	1.2336	1.0342	1.1872	1.4342
30	3.6245	2.5322	3.4521	2.9231	3.4325	3.3225	2.1221	2.0022	2.3211	2.2232
40	5.7845	4.2215	5.1431	5.1042	5.2511	5.4225	4.1932	4.0315	4.1928	4.2051

**Table 2:** PSNR and SSIM of noiseless CT images.

Input	Gaussian Noisy COVID-19 CT Image Dataset 1 (Average Results on 90 Images)							
	PSNR				SSIM			
	10	20	30	40	10	20	30	40
[10]	29.3544	27.4356	25.3446	22.3421	0.7344	0.6693	0.6133	0.5993
[11]	29.4434	27.4547	25.3426	22.23551	0.7888	0.6674	0.6196	0.5977
[12]	29.4425	27.3237	25.5667	22.3423	0.7466	0.6452	0.6104	0.5951
[13]	29.6446	27.2334	25.2468	22.3224	0.7343	0.6357	0.6124	0.5918
[14]	29.2336	27.3444	25.3458	22.7455	0.7354	0.6432	0.6124	0.5951
[15]	29.4547	27.5448	25.7436	22.5635	0.7548	0.6347	0.6154	0.5918
[16]	29.1237	27.4553	25.5633	22.4531	0.7355	0.6859	0.6194	0.5905
Proposed	30.0238	28.1339	25.1839	23.2239	0.7972	0.6993	0.6207	0.6021

**Table 3:** ED and DIV of noiseless CT images.

Input	Gaussian Noisy COVID-19 CT Image Dataset 1 (Average Results on 90 Images)							
	ED				DIV			
*Σ*	10	20	30	40	10	20	30	40
[10]	0.5821	1.3293	2.3448	3.4533	0.3541	1.4353	2.3443	4.3422
[11]	0.6838	1.3464	2.2346	3.2337	0.4438	1.4544	2.3422	4.2355
[12]	0.3864	1.5672	2.4564	3.4551	0.4424	1.3232	2.5666	4.3422
[13]	0.6828	1.1277	2.4664	3.3228	0.6448	1.2337	2.2464	4.3222
[14]	0.7864	1.4372	2.5664	3.2451	0.2334	1.3442	2.3456	4.7454
[15]	0.7828	1.5677	2.6434	3.3458	0.4548	1.5447	2.7433	4.5633
[16]	0.4855	1.6759	2.2354	3.5625	0.1235	1.4559	2.5632	4.4535
Proposed	0.2982	1.0694	2.0577	3.0121	0.0232	1.1334	2.1832	3.2236

**Table 4:** Visual analysis from the experts.

	[10]	[11]	[12]	[13]	[14]	[15]	[16]	Proposed
Edge Preservation	1	2	2	3	3	2	3	3
Noise Suppression	1	2	3	2	2	3	2	3
Artifact Removal	2	2	1	2	3	3	3	3
Heterogeneous Consistency	1	2	2	1	2	3	2	3
Data retention	2	1	2	2	2	2	2	3
Medical feature Extraction	3	3	2	3	2	3	3	3
Clarity/Sharpness	1	2	1	2	3	3	3	3

## Data Availability

Not applicable.
